# Supplementary material for: Development of quantitative high-throughput screening assays to identify, validate, and optimize small-molecule stabilizers of misfolded β-glucocerebrosidase with therapeutic potential for Gaucher disease and Parkinson’s disease
Source: bioRxiv. 2024 Mar 27:2024.03.22.586364. Preprint. [Version 1] doi: 10.1101/2024.03.22.586364 (PMC11071283; doi:10.1101/2024.03.22.586364)
Supplement: Supplement 3 [file NIHPP2024.03.22.586364v1-supplement-3.pdf]

bioRxiv preprint doi: https://doi.org/10.1101/2024.03.22.586364; this version posted March 27, 2024. The copyright holder for this preprint (which was not certified by peer review) is the author/funder. This article is a US Government work. It is not subject to copyright under 17 USC 105 and is also made available for use under a CC0 license.

Supplemental Figures:

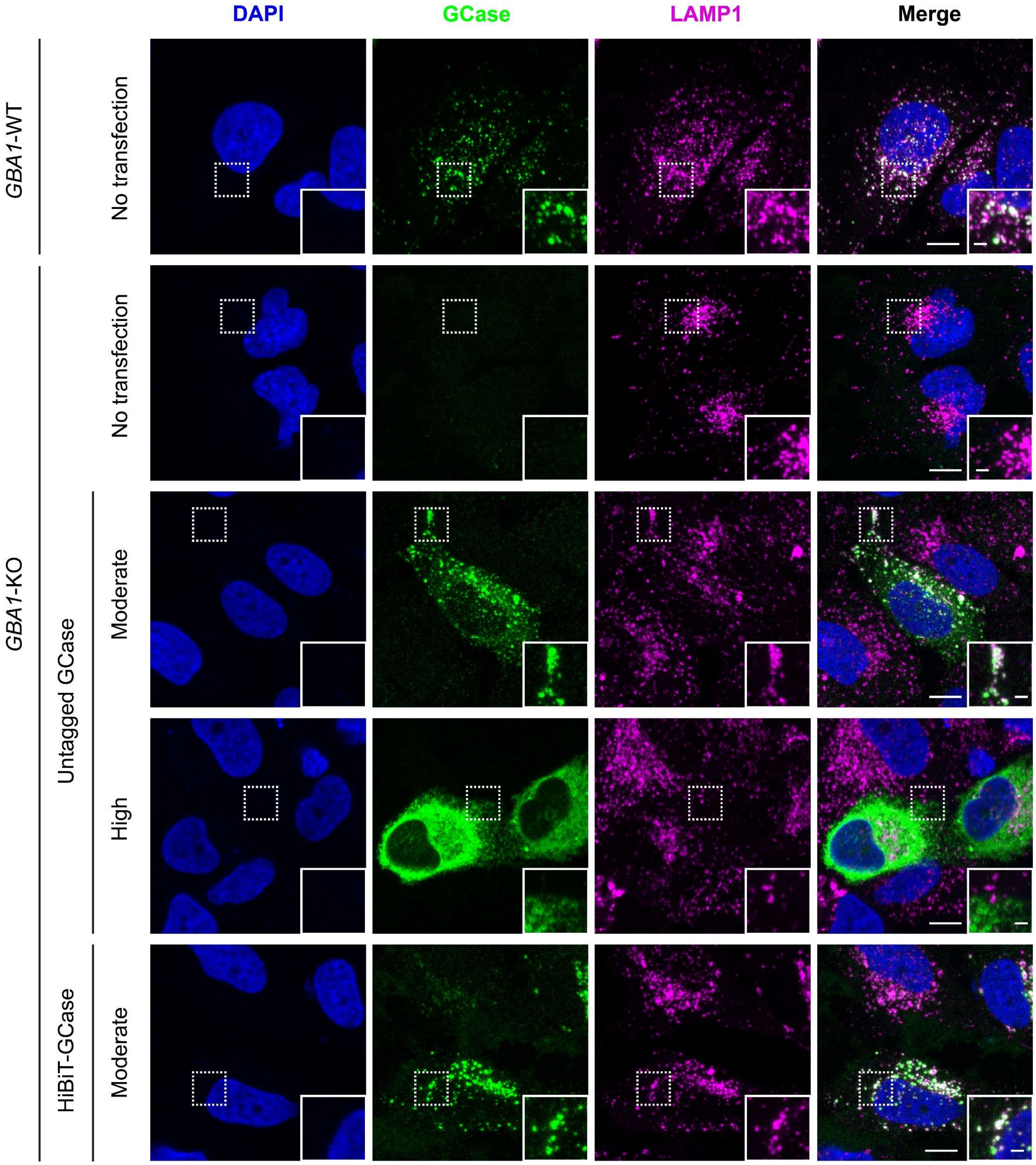

Figure S1. Overexpression of *GBA1* drives mis-localization. *GBA1*-WT or *GBA1*-KO H4 cells were transfected with constructs containing the HiBiT-*GBA1* transgene or untagged *GBA1*. Transfection produced a heterogenous population of cells with either low, moderate, or high levels of GCase expression; the latter perturbed GCase trafficking to the lysosome. GCase was stained with hGCase-1/23 antibody (green), while lysosomes were stained for LAMP1 (magenta).

bioRxiv preprint doi: https://doi.org/10.1101/2024.03.22.586364; this version posted March 27, 2024. The copyright holder for this preprint (which was not certified by peer review) is the author/funder. This article is a US Government work. It is not subject to copyright under 17 USC 105 and is also made available for use under a CC0 license.

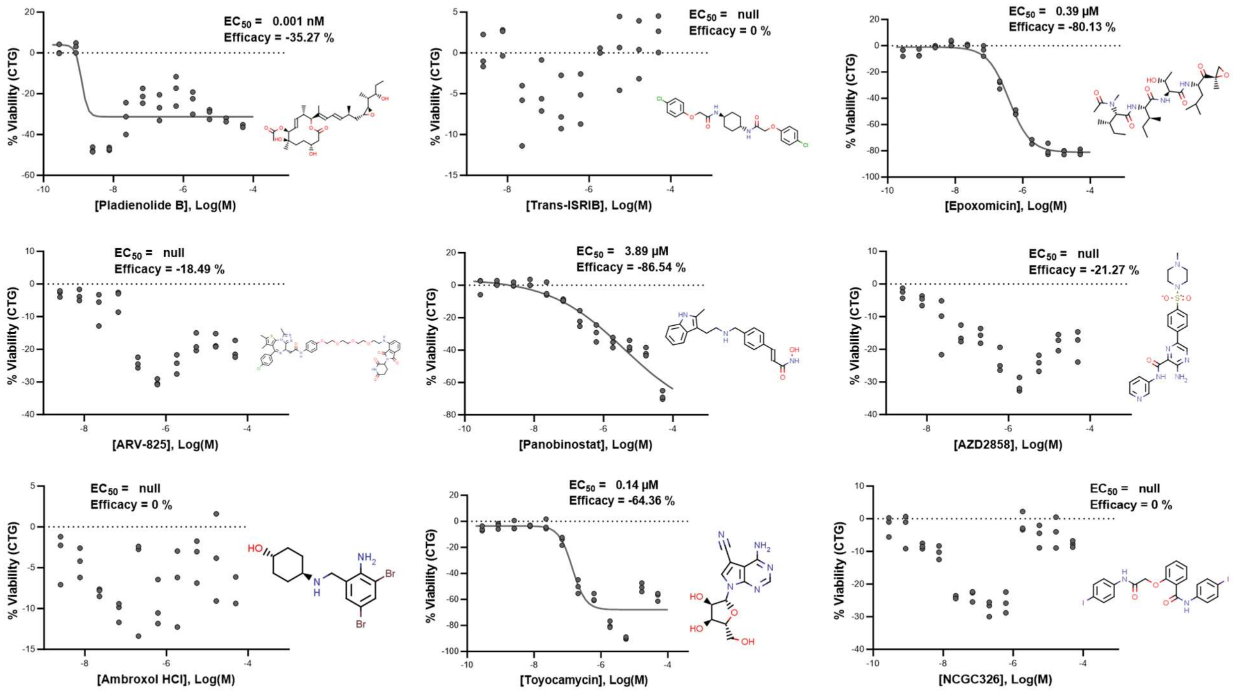

Figure S2. Follow-up cytotoxicity testing of selected primary qHTS hits using CellTiter-Glo assay. HiBiT-GCase-L444P H4 cells were seeded into 1536-well solid white plates (2,000 cells in 5 µL media) for 24 h and incubated with a titration of representative hits from the HiBiT primary screen for 24 h at concentrations ranging from 0.3 nM – 50 µM (12-point, 3x dilution series; *n* = 3). The CellTiter-Glo assay was then performed as described in the methods. Response values (% viability) are based on change in luminescence (RLU) in compound-treated versus DMSO-treated cells.

bioRxiv preprint doi: https://doi.org/10.1101/2024.03.22.586364; this version posted March 27, 2024. The copyright holder for this preprint (which was not certified by peer review) is the author/funder. This article is a US Government work. It is not subject to copyright under 17 USC 105 and is also made available for use under a CC0 license.

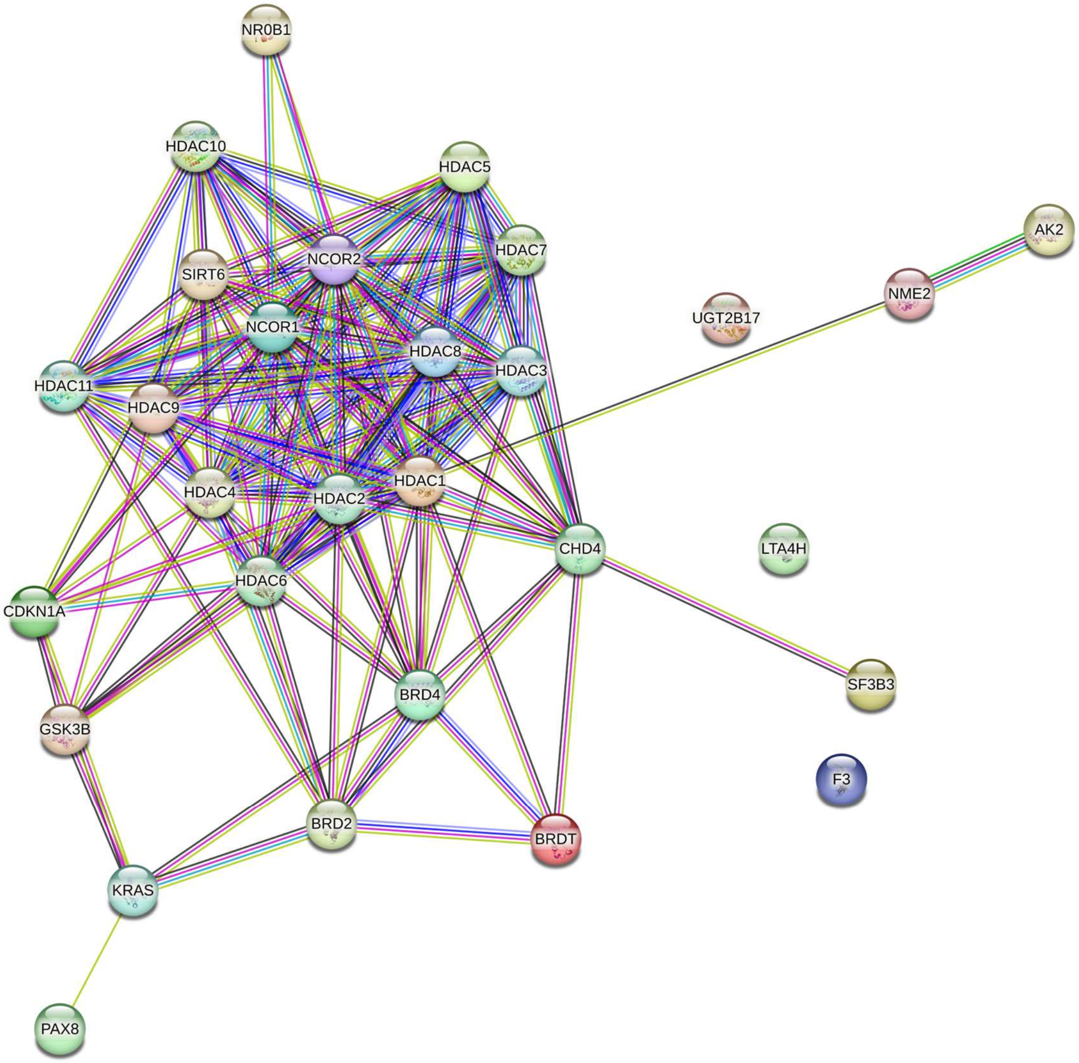

Figure S3. Interaction network of molecular targets of primary qHTS hits. Known and predicted protein-protein interaction network from STRING database for the 30 enriched targets identified from target profiles of final hit compounds. **Table S2** lists the pathways in the Reactome database that are overrepresented by these enriched targets, revealing potential pathways affected by the hit compounds.

bioRxiv preprint doi: https://doi.org/10.1101/2024.03.22.586364; this version posted March 27, 2024. The copyright holder for this preprint (which was not certified by peer review) is the author/funder. This article is a US Government work. It is not subject to copyright under 17 USC 105 and is also made available for use under a CC0 license.

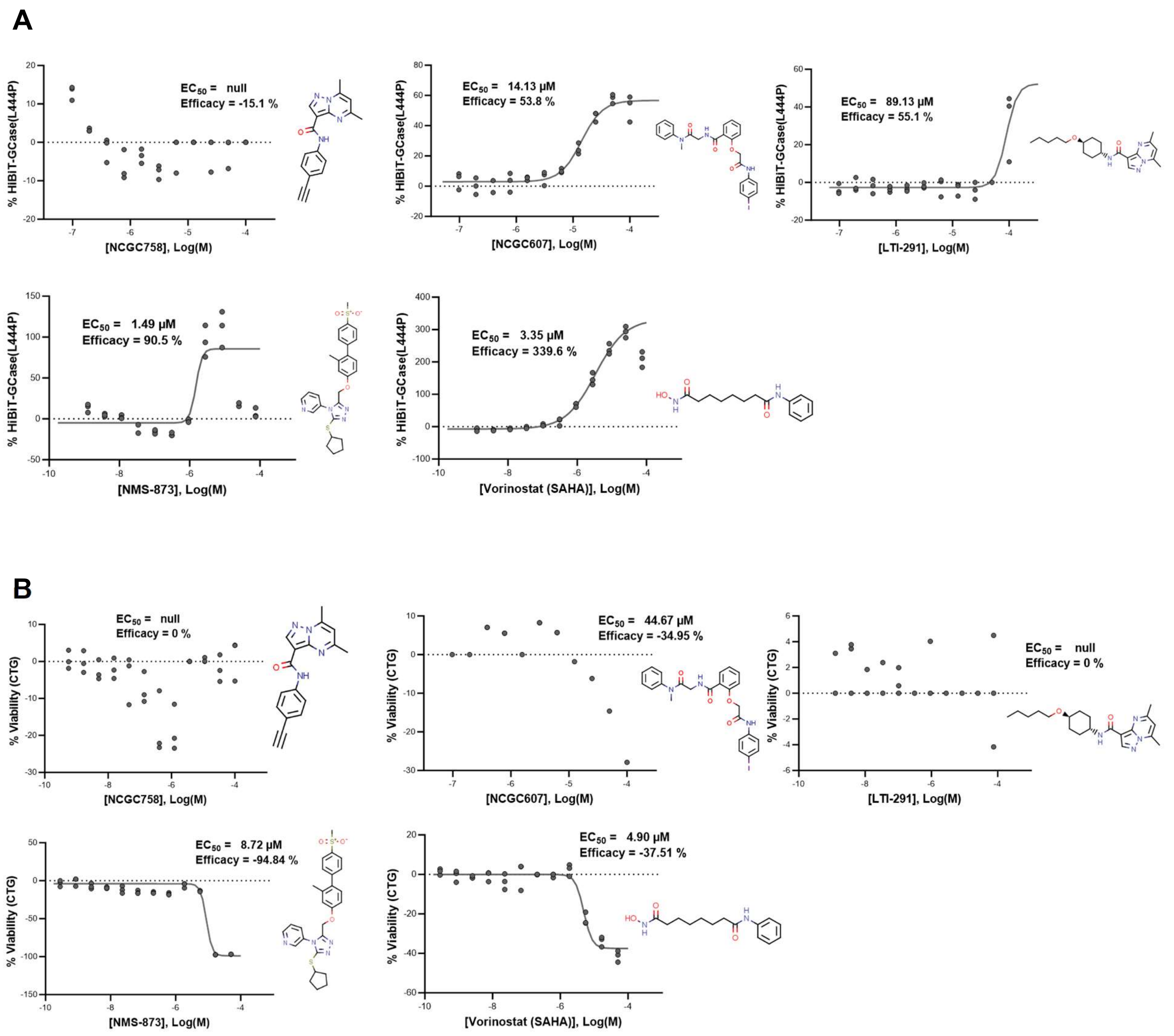

Figure S4. Primary qHTS curves and cytotoxicity testing for additional compounds. HiBiT-GCase-L444P H4 cells were seeded into 1536-well solid white plates (2,000 cells in 5 μL media) for 24 h and treated with a titration of NCGC758, NCGC607, LTI-291, NMS-873, and vorinostat (SAHA) for 24 h, after which either the HiBiT assay (**A**) or CellTiter-Glo assay (**B**) was performed as described in the methods. Data are from three independent replicates.

bioRxiv preprint doi: https://doi.org/10.1101/2024.03.22.586364; this version posted March 27, 2024. The copyright holder for this preprint (which was not certified by peer review) is the author/funder. This article is a US Government work. It is not subject to copyright under 17 USC 105 and is also made available for use under a CC0 license.

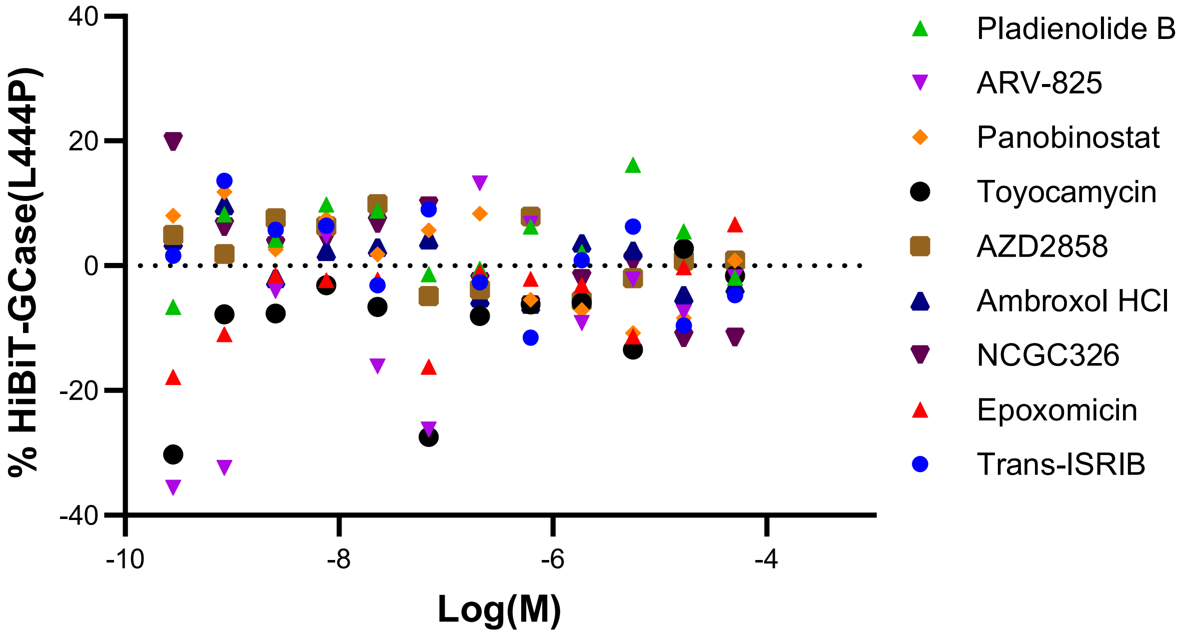

Figure S5. HiBiT-GCase-L444P assay interference testing for selected hits. Selected final hit compounds that increased HiBiT-GCase-L444P levels in H4 cells in the follow-up screen were tested for their ability to interfere with the reconstituted luciferase enzyme in the HiBiT-GCase assay. HiBiT-GCase-L444P H4 cells were seeded into 1536-well solid white plates (2,000 cells in 5 µL media) for 48 h and treated with a titration of compounds for 30 min, after which the HiBiT assay was performed to determine if the compounds were directly affecting HiBiT luminescence. Data are from three independent replicates.

bioRxiv preprint doi: https://doi.org/10.1101/2024.03.22.586364; this version posted March 27, 2024. The copyright holder for this preprint (which was not certified by peer review) is the author/funder. This article is a US Government work. It is not subject to copyright under 17 USC 105 and is also made available for use under a CC0 license.

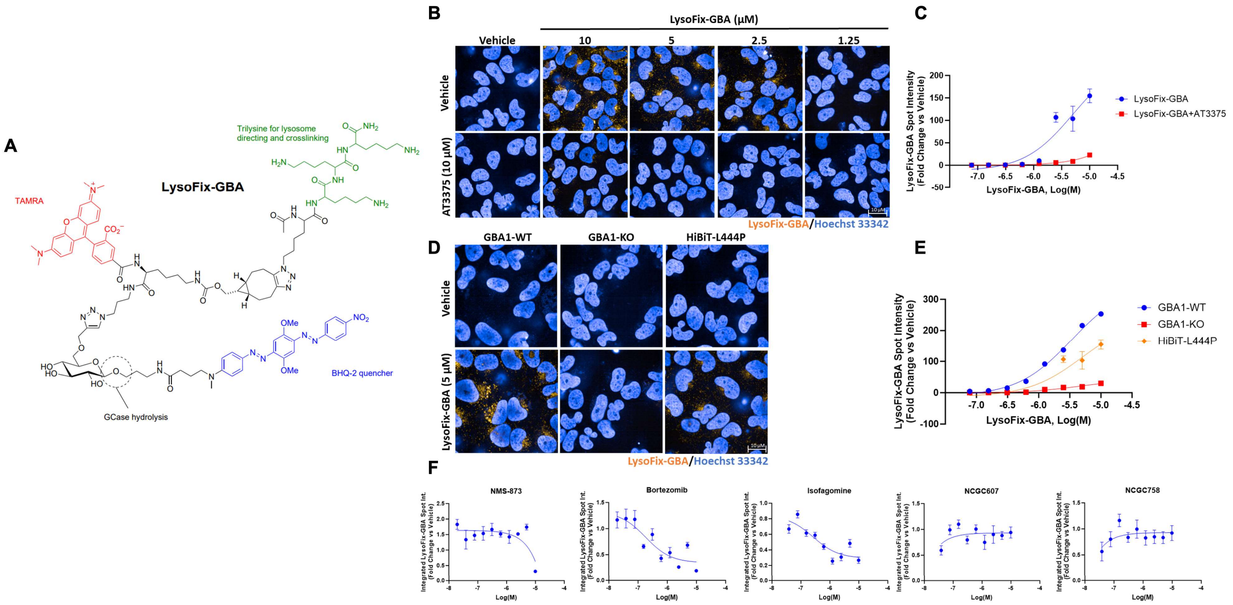

Figure S6. Implementation of LysoFix-GBA high-content screening assay in HiBiT-GCase-L444P H4 reporter line. (**A**) Chemical structure of LysoFix-GBA. (**B**) To optimize LysoFix-GBA concentration, HiBiT-GCase-L444P H4 cells were seeded into 384-well PerkinElmer PhenoPlates (25,000 cells in 40 µL media) for 24 h, followed by treatment with *GBA1* inhibitor AT3375 (10 µM) or vehicle (DMSO) for 24 h. Cells were then incubated with LysoFix-GBA (78 nM – 10 µM; 8-point, 2x dilution series) for 2 h at 37°C and imaged after 15 min of nuclear staining with Hoechst-33342 (1 µg/mL) in Fluorobrite media. (**C**) Data are represented as fold change in integrated LysoFix-GBA spot intensity per cell, relative to DMSO control. (Error bars: SEM [*n* = 4 – 6]). (**D, E**) Following the same approach, *GBA1*-WT, *GBA1*-KO, and HiBiT-GCase-L444P H4 cells were tested against a titration of LysoFix-GBA. (Error bars: SEM [*n* = 3]). (**F**) HiBiT-GCase-L444P H4 cells were treated with a titration of NMS-873, bortezomib, isofagomine, chaperone NCGC607, or chaperone NCGC758 for 24 h, and *GBA1* activity was assessed by LysoFix-GBA (5 µM). (Error bars: SEM [*n* = 4 – 6]).

bioRxiv preprint doi: https://doi.org/10.1101/2024.03.22.586364; this version posted March 27, 2024. The copyright holder for this preprint (which was not certified by peer review) is the author/funder. This article is a US Government work. It is not subject to copyright under 17 USC 105 and is also made available for use under a CC0 license.

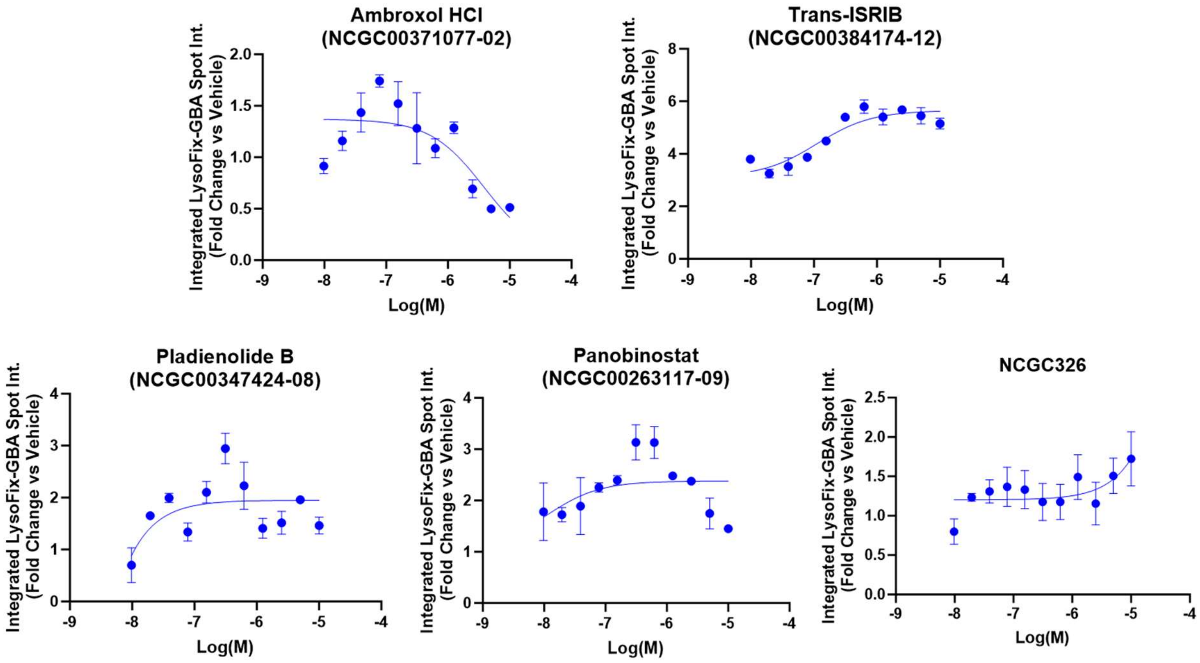

Figure S7. Evaluation of hit compounds with LysoFix-GBA assay after 72 h of compound incubation. HiBiT-GCase-L444P H4 cells were seeded into 384-well PerkinElmer PhenoPlates (25,000 cells in 40 µL media) and incubated for 24 h. Thereafter, the cells were treated with a titration of compounds (9.8 nM – 10 µM; 11-point, 2x dilution series) for 72 h and then incubated with LysoFix-GBA (5 µM) for 2 h at 37°C and 5% CO_2. High-content imaging was performed after 15 min of nuclear staining with Hoechst-33342 (1 µg/mL) in Fluorobrite media. Data are represented as the fold change (compound-treated vs. DMSO-treated) in integrated LysoFix-GBA spot intensity per cell. Dose-response curves were fit using log(agonist) vs. response (three parameters). (Error bars: SEM [n = 2 – 5]).

bioRxiv preprint doi: https://doi.org/10.1101/2024.03.22.586364; this version posted March 27, 2024. The copyright holder for this preprint (which was not certified by peer review) is the author/funder. This article is a US Government work. It is not subject to copyright under 17 USC 105 and is also made available for use under a CC0 license.

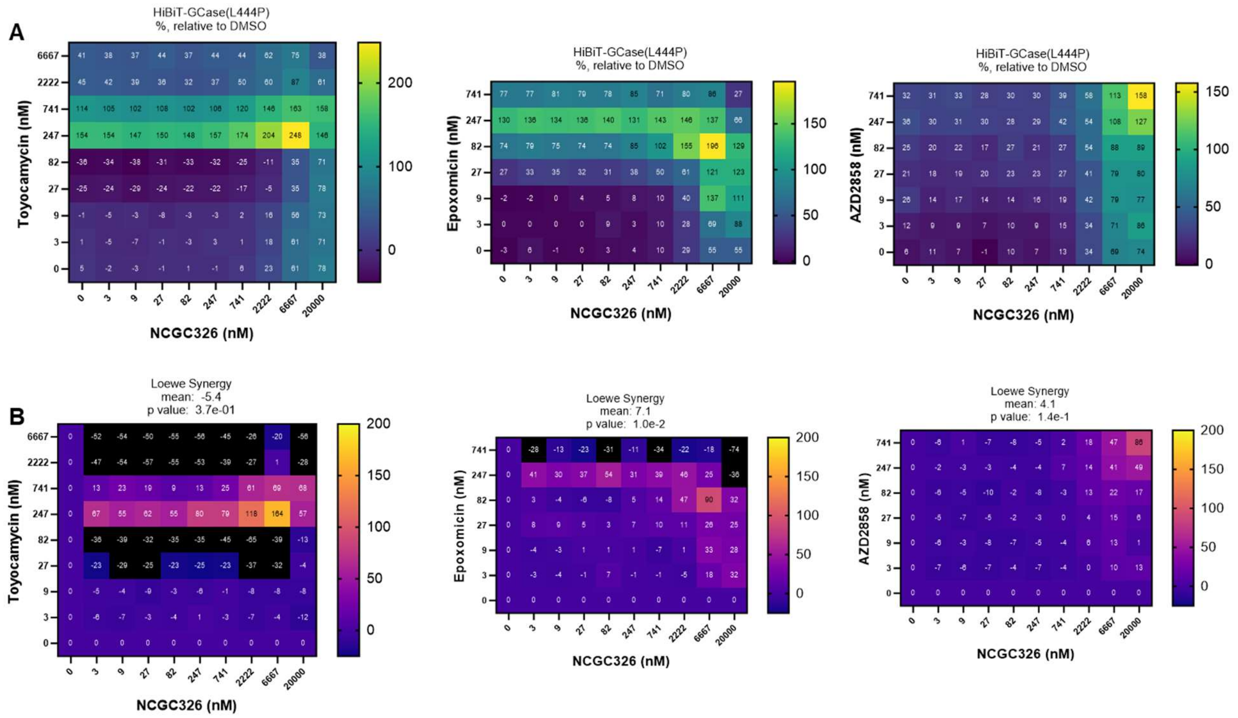

Figure S8. Matrix combination screening with NCGC326 and additional hits from primary HiBiT screen. HiBiT-GCase-L444P H4 cells were tested in 10 x 10 pairwise dose-response combinatorial matrix format. Cells were treated for 24 h with chaperone NCGC326 in a 9-point titration (3 nM – 20 µM, 3x dilution) against the same 9-point titration of toyocamycin, epoxomicin, or AZD2868; the HiBiT-GCase lytic assay was then performed. Luminescence response values were normalized to intraplate DMSO-treated controls, such that 100% activity reflects a doubling of HiBiT-GCase levels. Synergy was evaluated based on the dose-response matrix (**A**) and the Loewe synergy score (**B**). In general, negative, zero, and positive synergy scores indicate antagonistic, additive, and synergistic interactions, respectively, between drugs. If a dose-response curve could not be fit due to toxicity at top concentrations, these concentrations were omitted from the analysis. *n* = 3.
